# The sweet taste quality is linked to a cluster of taste fibers in primates: lactisole diminishes preference and responses to sweet in S fibers (sweet best) chorda tympani fibers of *M. fascicularis *monkey

**DOI:** 10.1186/1472-6793-9-1

**Published:** 2009-02-18

**Authors:** Yiwen Wang, Vicktoria Danilova, Tiffany Cragin, Thomas W Roberts, Alexey Koposov, Göran Hellekant

**Affiliations:** 1 Department of Physiology and Pharmacology, Medical School, University of Minnesota-Duluth, 1035 University Dr, Duluth, MN 55812, USA

## Abstract

**Background:**

Psychophysically, sweet and bitter have long been considered separate taste qualities, evident already to the newborn human. The identification of different receptors for sweet and bitter located on separate cells of the taste buds substantiated this separation. However, this finding leads to the next question: is bitter and sweet also kept separated in the next link from the taste buds, the fibers of the taste nerves? Previous studies in non-human primates, *P. troglodytes, C. aethiops, M. mulatta, M. fascicularis and C. jacchus*, suggest that the sweet and bitter taste qualities are linked to specific groups of fibers called S and Q fibers. In this study we apply a new sweet taste modifier, lactisole, commercially available as a suppressor of the sweetness of sugars on the human tongue, to test our hypothesis that sweet taste is conveyed in S fibers.

**Results:**

We first ascertained that lactisole exerted similar suppression of sweetness in *M. fascicularis*, as reported in humans, by recording their preference of sweeteners and non- sweeteners with and without lactisole in two-bottle tests. The addition of lactisole significantly diminished the preference for all sweeteners but had no effect on the intake of non-sweet compounds or the intake of water. We then recorded the response to the same taste stimuli in 40 single chorda tympani nerve fibers. Comparison between single fiber nerve responses to stimuli with and without lactisole showed that lactisole only suppressed the responses to sweeteners in S fibers. It had no effect on the responses to any other stimuli in all other taste fibers.

**Conclusion:**

In *M. fascicularis*, lactisole diminishes the attractiveness of compounds, which taste sweet to humans. This behavior is linked to activity of fibers in the S-cluster. Assuming that lactisole blocks the T1R3 monomer of the sweet taste receptor T1R2/R3, these results present further support for the hypothesis that S fibers convey taste from T1R2/R3 receptors, while the impulse activity in non-S fibers originates from other kinds of receptors. The absence of the effect of lactisole on the faint responses in some S fibers to other stimuli as well as the responses to sweet and non-sweet stimuli in non-S fibers suggest that these responses originate from other taste receptors.

## Background

A series of elegant studies in genetically modified mice show that sweet and umami tastes are dependent on T1R-receptors, that bitter taste is caused by stimulation of T2R receptors, that these two receptors never are found in the same taste receptor cell (TRC) and that the TRC determines the behavioral response [1-7]. One study, for example, showed "that mice engineered to express a bitter taste receptor in 'sweet cells' become strongly attracted to its cognate bitter tastants, whereas expression of the same receptor (or even a novel GPCR) in T2R-expressing cells resulted in mice that are averse to the respective compounds" [5]. The authors concluded that the taste receptor cells trigger intake behavior [5].

The above-mentioned discovery of a unique set of taste receptors for the sweet and bitter taste qualities has provided one answer to the long lasting question on how sweet or bitter taste is created on the tongue. However, it has not solved the problem on how the information from the sweet and bitter receptor bearing taste cells is coded in the taste nerves?

The first suggestion that each of the human taste qualities is related to a particular type of taste fiber was based on recordings of the chorda tympani (CT) and glossopharyngeal (NG) nerves of cat [8]. It was in many ways a seminal study and presented several observations that later studies confirmed. For example, it identified that different taste fibers respond to different taste qualities and noted that the NG nerve contains a larger proportion of mechanosensitive fibers than the CT. It also correctly connected a lack of response to sucrose with the inability of cats to appreciate sucrose. The reason for this was recently elucidated [9]. The sweet sensitive taste fibers were later discovered in dog [10].

Although the relationship between animal taste fibers and human taste qualities was strengthened by recordings of rhesus monkeys [11,12], investigators recording from non- primates found a weak relationship between human taste qualities and types of taste fibers. It is likely that the less than perfect parallel between rodent data and human taste qualities is the reason why the idea that each taste quality is conveyed in a unique group of taste fibers is not universally accepted and was probably one of the reasons why the across-fiber pattern was presented as an explanation of how tastes are coded [13]. According to this theory, every taste fiber contributes to every taste sensation [14-16]. One important consequence of this is that, whereas textbooks of Physiology detail the different TRCs and there specific receptors, there is little or nothing mentioned on the relationship between the responses from the taste receptor specific TRCs and the taste fibers, that is, how taste is coded in peripheral nerves.

One way to demonstrate if there is a connection between a taste quality and a specific group of taste fibers (that does not include any other fiber groups) is to apply a compound that changes or abolishes one of the taste qualities, and then study the accompanying changes in taste fibers. Lactisole is such a compound, because in humans it suppresses the sweet tastes of sugars and artificial sweeteners [17], but has no effect on the perception of bitterness, sourness and saltiness [18,19].

Here we report that lactisole, at concentration used in humans, in *M. fascicularis *diminished its preference for sweet and decreased the response of sweeteners in its S fibers without affecting its behavioral response to non-sweet compounds or the response in any other taste fiber type. These data present further support for our hypothesis that the taste of sweeteners is conveyed by S fibers and that the sweet taste quality is linked to this particular group of taste fibers.

## Methods

### Animals and stimuli

Behavioral and electrophysiological data were obtained from 5 female *M. fascicularis*, weighing 2.1–2.4 kg. Table 1 presents the compounds and concentrations used in the electrophysiological and behavioral experiments. We also used a second set of the same compounds mixed with 1.25 mM lactisole. These are not listed in Table 1.

**Table 1:** Stimuli Used in Experiments

Compound	Electrophysiological	Behavioral
Ace-K	3.5 mM	1.5 mM
Alitame	0.3 mM	
Ascorbic acid	40 mM	40 mM
Aspartame	5 mM	0.5 mM
Aspartic acid	50 mM	
Caffeine	100 mM	
Citric Acid	50 mM	50 mM
Ethanol	3000 mM	
Lactisole	1.25 mM	1.25 mM
MSG	70 mM	
NaCl	100 mM	500 mM
Saccharin	1.6 mM	0.1 mM
SC45647	0.1 mM	0.04 mM
SOA	1 mM	
Stevioside	0.9 mM	
Sucrose	300 mM	50 mM
WT Brazzein	0.015 mM	
Xylitol	800 mM	120 mM
QHCl	5 mM	20 mM

### Behavioral experiments

The animals were individually housed and had access to water throughout the behavioral tests. We utilized the two-bottle method (TBP). First, the animals went through a training period during which a graded cylinder with sucrose was left on each cage. When the animals consistently drank from the bottle, we switched to two graded cylinders on the cage, one cylinder contained 50 ml water, the other 50 ml of sucrose. During this training period the animals learnt to sample the cylinders, whose left or right position was shifted at each occasion. In the next phase the sucrose was replaced with one of the sweeteners shown in Table 1. Since macaques like sweet, the animals rapidly learnt to sample the cylinders and consumed the sweeteners avidly. The training period was followed by tests of the effect of lactisole on the intake of these compounds. Then one cylinder contained the sweetener, while the other contained the same sweetener with 1.25 mM lactisole added. The tests were conducted in duplicate to verify results. We also compared the intake between water and 1.25 mM lactisole. These tests were conducted once a day for 15 minutes.

Data on the effect of lactisole on the intake of non-sweet compounds were also obtained by comparing the intake of the same compound with and without lactisole. During the tests with the non-sweet compounds the bottles were left on the cage for 1–2 h, as otherwise no solution would be consumed. The significance of the differences between the behavioral data obtained with and without lactisole was determined with t-tests: paired two samples for means with 90% confidence.

### Surgery

The electrophysiological data were obtained from the right chorda tympani proper (CT) of the same animals as in the behavioral experiments. The anesthesia was initiated with i.m. ketamine, 50 mg/animal. The monkey was then intubated and the anesthesia maintained with halothane (0.7–1.0%). Fluid losses were replaced with 5% dextrose and lactated Ringer's solution through an i.v. cannula. Body temperature, heart and respiratory rates, CO_2 _in expired air, and O_2 _in blood were continuously monitored and recorded. The method to dissect the right CT has been described several times e.g., [20]. In short, an incision was made along the mandibular angle between the rostral lobes of the parotid gland and the mandibular bone. Then the tissue attached to the mandibular angle was dissected through and the caudo-medial side of the pterygoid muscle followed down to its origin at the pterygoid plate of the skull to the CT. The nerve was freed from its junction with the lingual nerve to a point close to the bulla tympani where it is covered by venous sinuses in most cases cf. [21].

### Stimulation

The tastants were delivered to the tongue with an open flow system (Taste-O-Matic), controlled by a computer and custom made software. It delivered the solutions at given intervals, over a preset time, under conditions of constant flow and temperature (33°C) [20]. The stimulation time was chosen to be long enough to elicit a clear taste response, but as short as possible to obtain as much data as possible, since one never knows when a single fiber may fade. Usually 5 sec stimulation was applied. Between stimulations the tongue was rinsed for 55 to 52 sec with artificial saliva described in [23]. The rinsing time was chosen to minimize cross-adaptation between stimuli. We also never applied two stimuli representing the same taste quality after each other. It should also be noted that the switch from rinse to stimulus and back to rinse was accomplished without any mechanical or temperature artifacts. As a control we repeated stimulation with NaCl, sucrose, QHCl and acid more than once during a cycle.

### Electrophysiology; Recording impulses from more than one single fiber in the same filament

The nerve impulses were recorded with an isolated differential amplifier and fed into an electrostatic recorder, displayed on an oscilloscope and the computer via a data acquisition card, which digitized the signal at a rate of 50 kHz with a 12 bit analog-to-digital converter. We used Recorder software (Plexon, Inc.) to set up the data acquisition channels, monitor the signals, control the data recording process and save the whole raw neural signal and binary coded stimuli parameters with time marks. The data were then imported into Offline Sorter (OFS, Plexon, Inc) and NeuroExplorer (Plexon, Inc) for spike sorting and further analysis. The sorting method is based on the feature analysis method, which, using a sophisticated cluster analysis algorithm, separated waveforms according to their shapes and firing patterns. Our approach allowed us to obtain responses of 40 CT fibers in 5 monkeys.

Analysis of the spike trains included building of time histograms, numerical analysis and scoring of the spontaneous activity and responses to different compounds. As an extra control, we built histograms of the neuronal activity throughout the recording to estimate consistency of the spike trains and fiber responsiveness. The response measure usually used in single fiber recordings is numbers of spikes per second over the stimulation period. The spontaneous activity before a stimulation was deducted from the activity during stimulation.

To detect if there is an organization of the taste fibers, we used hierarchical cluster analysis (SYSTAT). It is a multivariate procedure for detecting natural groupings in data. The responses to all stimuli were taken into consideration and the analysis considered each stimulus as an independent variable and calculated Pearson correlation coefficients between response profiles. We used correlation measures, because they are not influenced by differences in the absolute values of the responses. The whole matrix of the correlation coefficients was subjected to the analysis and we looked for similarities between whole pattern of response profiles. We used an average linkage method. The result was presented as a dendrogram.

Responses of fibers belonging to the same cluster were first evaluated by two-way ANOVA on ranked data. Differences between cluster's responses with and without lactisole were assessed using t-test. For all tests P < 0.05 were considered significant.

The four basic stimuli, NaCl, citric acid, QHCl, and sucrose, were also used to categorize each fiber by its best stimulus. The breadth of tuning (H) was calculated according to the formula [22].

## Results

### Behavior

**Figure 1:**
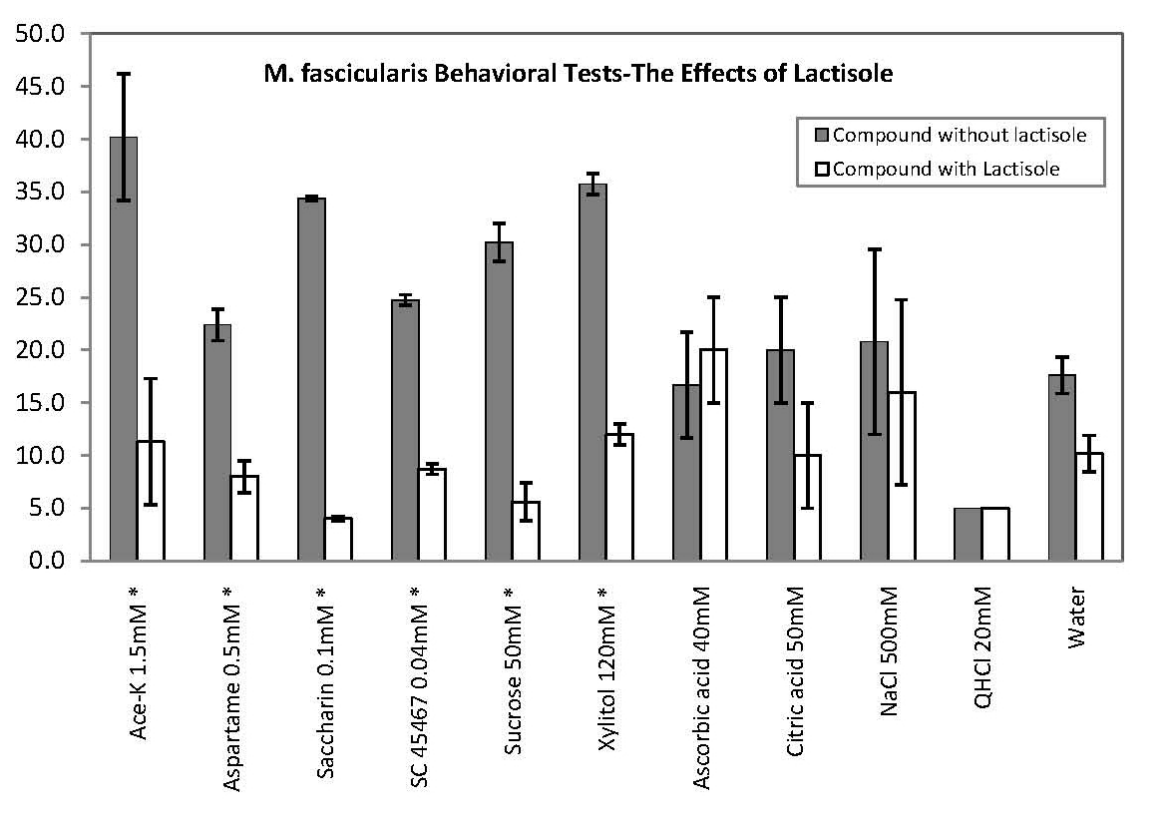
**Results of two-bottle preference tests with one bottle with the tastant and the other with 1.25 mM lactisole added to the tastant**. Each bottle contained 50 ml. It is evident that presence of lactisole made the sweetener less attractive, but had no significant effect on the intake of the non-sweet compounds. Error bars SE. The asterisks* signifies a significant difference in intake.

Figure 1 shows the result of the two-bottle preference (TBP) tests. The left staples in each pair display the average consumption of the compound without lactisole and the right ones with lactisole. Asterisks denote significant difference of intake at 90% confidence limits.

It is evident that lactisole in the sweeteners significantly lowered the intake, while it had no effect on the non-sweet compounds. Its largest effect was on the intake of saccharin and sucrose, followed by about equal effect on acesulfame-K, xylitol, aspartame and SC 45647. There was no significant difference in intake between water and 1.25 mM lactisole in water or any of the non-sweet compounds with and without lactisole. Thus the difference in intake was only significant for the sweeteners.

### Electrophysiology

**Figure 2:**
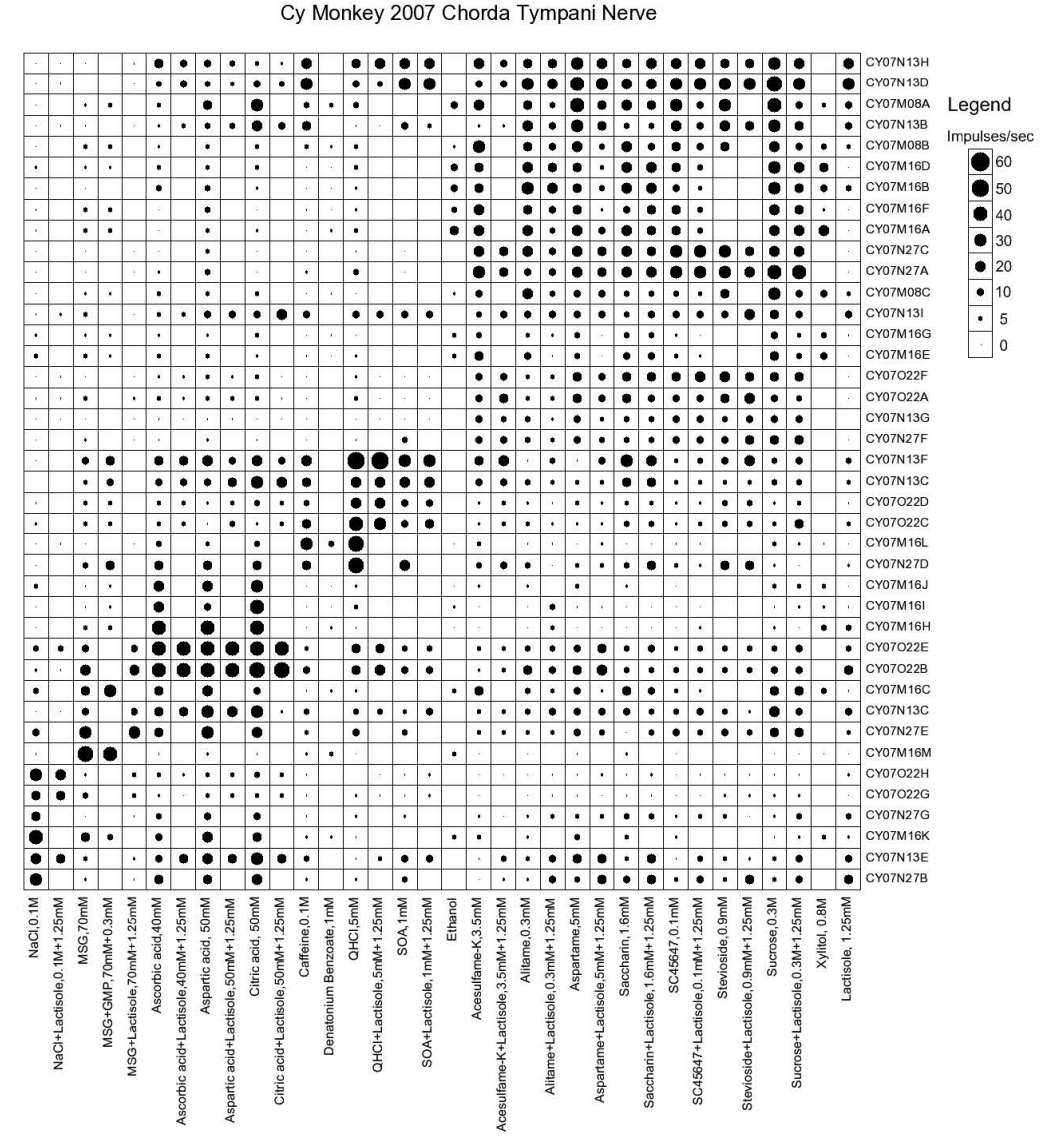
**An overview of the response profiles of 40 single CT taste fibers with the use of a topographical method**. The area of the dots represents impulse activity per sec over 5 sec of stimulation. Absence of mark shows that data are missing. The stimuli were arranged along the x-axis in order of salty, sour, bitter and sweet and along the y-axis in groups: NaCl (N fibers), acids (H fibers), bitter (Q fibers) and sucrose best fibers (S fibers). Every second column showed the response to the sweetener with lactisole added.

**Figure 3:**
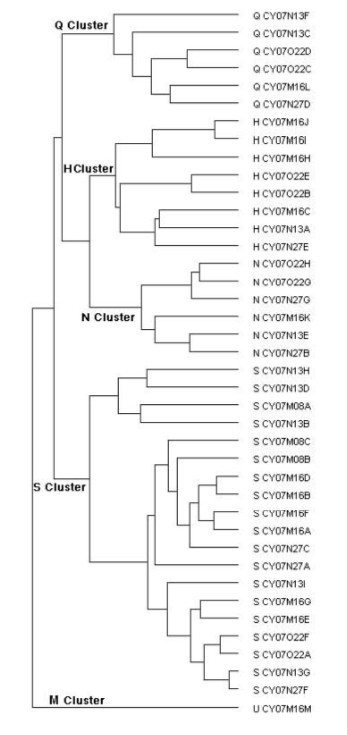
**Results of hierarchical cluster analyses of the response profile of 40 CT taste fibers**. Intercluster similarity was measured with the Pearson correlation coefficient, and the cluster analysis proceeded according to the average linkage method. Number of response categories of the fibers on the basis of their responses to the basic solutions is listed on the right. Q, H, N, S and M stand for QHCl-, citric acid-, NaCl, sucrose-, MSG-best fibers.

Figure 2 presents an overview of the response in each individual fiber with its identity along the vertical axis and stimulus listed along the horizontal. The stimuli were arranged along the X axis in order of salty, umami (MSG and MSG with GMP), sour, bitter and sweet, and the fibers along the Y axis in groups of NaCl- (N cluster), MSG-, citric acid- (H cluster), QHCl- (Q cluster) and sucrose-best (S cluster) as shown in Figure 3. The area of each dot in Figure 2 represents the impulse activity over the first 5 sec of stimulation minus spontaneous activity before each stimulation. Absence of a dot shows that data are missing. Every second column shows the response to the tastant mixed with 1.25 mM lactisole.

The N cluster consisted of fibers that responded best to NaCl. Three of the fibers showed also a response to the acids and two of these fibers were clearly also stimulated by lactisole and to some extent by the sweeteners. It is likely that their response to sweetener/lactisole originates mostly from the effect of lactisole. Their breath of tuning (H), which gives a numerical value on how specific a group of fibers is, was 0.64, SD 0.05.

The H cluster consisted of acid-best fibers but five fibers responded also to the MSG stimuli. Breath of tuning was 0.63, SD 0.1, which indicates that they were about as specific as the fibers in the previous cluster.

The Q cluster fibers were predominantly responding to QHCl and to SOA, although acids and three sweeteners, saccharin, stevioside and acesulfame-K, gave a response in two fibers. This may by explained by the observation that the taste of both saccharin and acesulfame-K includes a bitter component and stevioside has an additional licorice taste. As a group the Q fibers were slightly more specific than the previous fibers (H = 0.55, SD 0.05).

**Figure 4:**
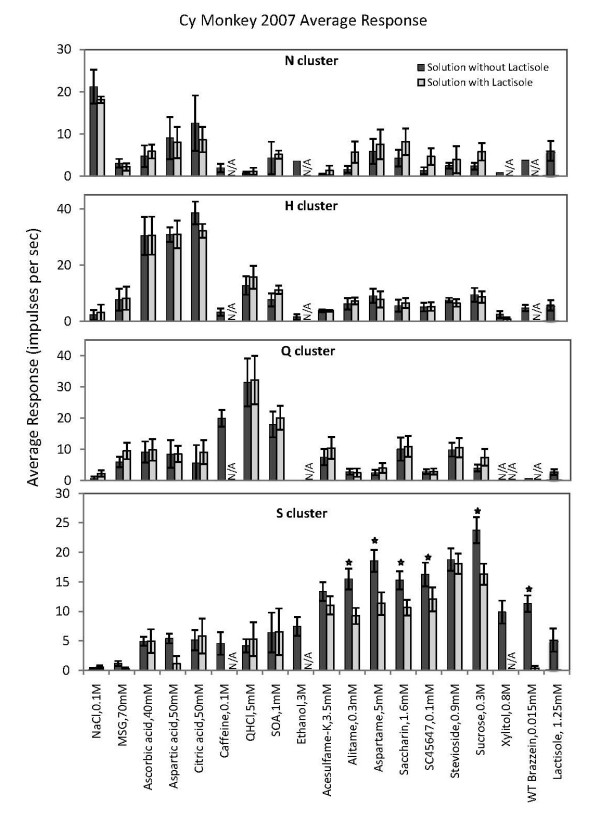
**Average response profiles of from the top the N cluster, H cluster, Q cluster and S cluster**. Error bars are SE. Dark columns, the tastants without lactisole, open columns, tastants with 1.25 mM lactisole added. Asterisks denote a difference between the two columns at a significance level of > 90%. Only the responses to sweeteners in the S fiber cluster were significantly suppressed by the addition of lactisole.

The S fibers were more specific than any other (H = 0.4, SD 0.06) although the dendrogram suggests that these fibers could be composed of three subgroups of which the upper one consisting of 4 fibers was less specific than the other two. Although it may not be visible from Figure 2, in all 19 S fibers the response to sucrose with lactisole was smaller than to sucrose alone. This is further shown in Figure 4.

### Dendrogram

Figure 3 presents the results of hierarchical cluster analysis of the 40 single taste fibers. We used the responses to all stimuli without lactisole. Listed on the left of the dendrogram is each fiber's response category and on the right its identity number. The cluster analysis clearly separated N, H, S and Q clusters. The analysis identified 6 fibers in the N fiber cluster, 8 H fibers, 6 Q fibers and 19 S fibers.

As shown in Figure 2, fiber CY07N13E responded better to citric acid than NaCl. Therefore one might suspect that it is falsely classified into the N cluster. However, the cluster analysis only calculates the Euclidean distance between the fibers and because the result is not normalized, this fiber is closer to the N fibers than to any other group. In addition, the analysis placed another fiber (U CY07M16M) in an additional fifth cluster, which we labeled M based on its response to MSG alone or mixed with GMP. It was unique in its singular response to the stimuli representing the umami taste quality, MSG and MSG with GMP.

### Effects of lactisole

Figure 4 shows the effect of addition of lactisole on the responses of each of the above clusters. Only data of fibers tested with the same stimulus with and without lactisole are included. Thus, the left column in each pair shows the response to the tastant alone, while the right column displays the response when lactisole had been added to the stimulus. Error bars are SE.

The plot shows that addition of lactisole did not significantly suppress the response to any stimulus in the N cluster. Lactisole in itself gave a response in the N fibers that may explain the increase of the response to some of the tastants with lactisole. In the H and Q cluster lactisole did not change the responses to any stimuli.

The data from the S cluster shows three features, relevant to the hypothesis that S fibers convey sweet taste. First, all S fibers responded to all sweeteners tested. This indicates that they received input from the same receptor type. Second, lactisole suppressed the response of all sweeteners, although the effect was not significant in acesulfame-K and stevioside. On the average the responses of the lactisole containing sweeteners was 70%, SD 6, of the responses of stimulation with the sweetener alone. Third, lactisole did not suppress the responses of the non-sweet compounds. Thus, its effect was limited to the responses of sweeteners in S fibers.

### Effect of lactisole on temporal pattern or temporal intensity

**Figure 5:**
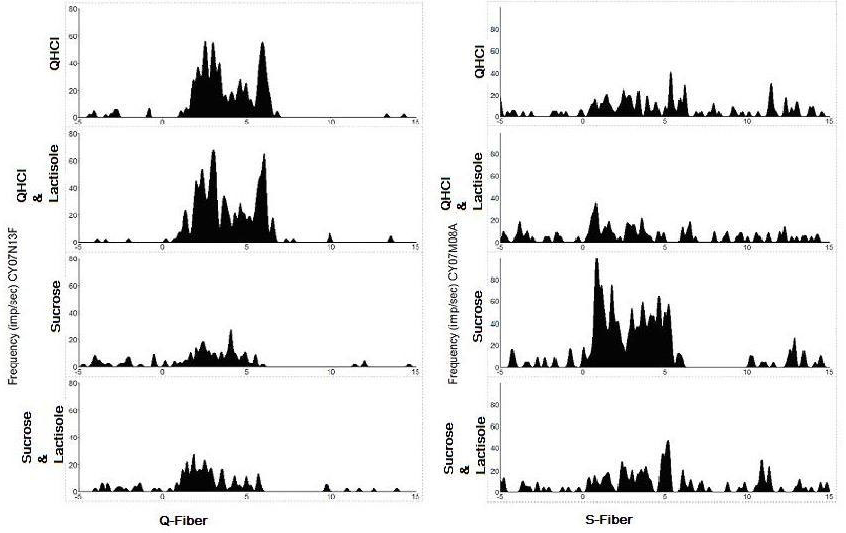
**It presents two examples of the effect of lactisole on the temporal pattern also called time intensity (TI) of the responses of a non-S fiber (Q fiber CY07N13F) and an S fiber (CY07M08A)**. The impulse activity of each fiber is displayed during 5 sec of rinsing with artificial saliva, followed by 5 sec of stimulation and then by 10 sec of renewed rinsing with the artificial saliva. The upper trace in each pair shows the response without lactisole and the lower trace the response with lactisole added to the stimulus. Only in the S fiber the response to sucrose was suppressed by lactisole.

Figure 5 presents two examples of the effect of lactisole on the temporal pattern or time intensity (TI) of a non-S fiber (Q fiber CY07N13F) and an S fiber (CY07M08A). The impulse activity of each fiber is displayed during 5 sec of rinsing with artificial saliva, followed by 5 sec of stimulation and then by 10 sec of renewed rinsing with the artificial saliva. The upper trace in each pair shows the response without lactisole and the lower trace the response with lactisole added to the stimulus.

Several features should be noted. First, while sucrose gave a large response in the S fiber and QHCl in the Q fiber, QHCl also elicited a faint response in the S fiber and sucrose in the Q fiber. Second, only the response to sweet in the S fiber was diminished by lactisole addition; there was no effect by lactisole on the response to QHCl or sucrose in the Q fiber or by lactisole on the response to QHCl in the S fiber. Third, the suppression of the sucrose response of the S fiber was visible in the nerve activity during the whole stimulation period and not only in part of it, for example, in the phasic or the tonic part.

### Multidimensional scaling

**Figure 6:**
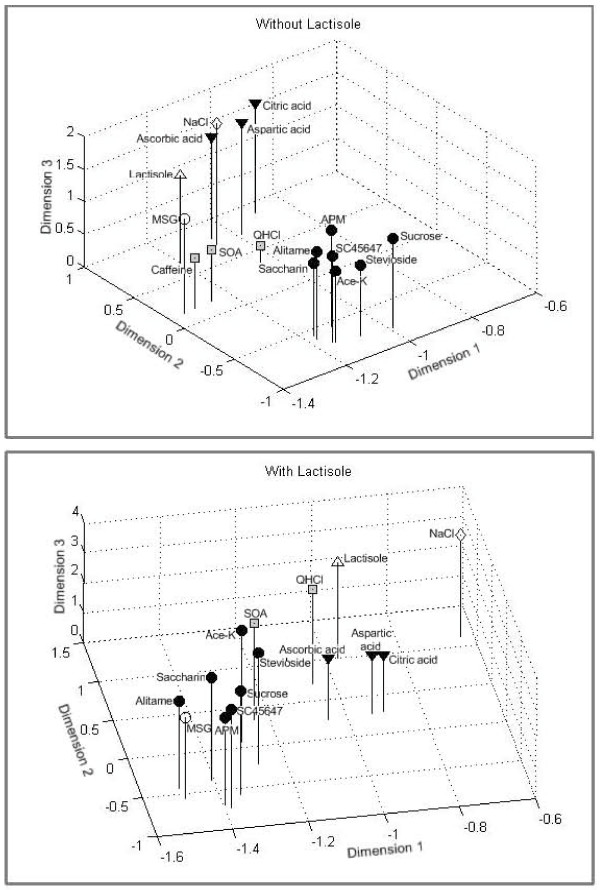
**Distribution of 16 tastants in a 3-D space resulting from multidimensional scaling**. The distribution was calculated with Pearson correlation coefficient between stimuli across 40 CT fibers. The stress value is 0.047. The top plot shows that the sweeteners before lactisole formed a tight group further away from the non-sweet stimuli than after lactisole. The results suggest that lactisole diminishes the taste difference between sweeteners and other tastants.

Based on a correlation matrix of the stimuli, we performed multidimensional scaling for compounds without and with lactisole. The spatial representation of the similarities among 16 stimuli without lactisole is shown in the upper diagram of Figure 6. The stress value is 0.047. The top plot shows that the sweeteners before lactisole formed a tight group. The lower plot in Figure 6 shows that adding lactisole to the compounds shrunk the distance between the sweet group and the non-sweet group. This suggests that the nerve response to sweet was less different from that of non-sweet compounds when the sweeteners contained lactisole. The interpretation could be that lactisole diminished the taste difference between sweet and non- sweet tastants.

## Discussion and conclusion

The results of Figure 1 demonstrated that lactisole suppressed the intake of sweeteners but had no effect on intake of non-sweet tastants or water. The hierarchical cluster analysis used objective statistical methods to classify the taste fibers according to their responses in 5 clusters shown in Figure 3. One cluster consisted of S fibers, which responded to sweet compounds. Comparison in S-fibers between the responses of sweet stimuli with and without lactisole showed that lactisole suppressed the response to sweeteners but had no effect on other stimuli or responses in other types of taste fibers (Figure 2 and 4).

In the following these results and conclusions will be discussed within the context of data from more than 20 earlier studies. We will discuss:

a. Possible influence of diet on proportions of taste fiber types in two macaques

b. Comparison with earlier data obtained with the sweet taste modifiers miraculin and gymnemic acid (GA)

c. The parallel between lactisole effects in homo and M. fascicularis

d. The relationship between the T1 receptors, lactisole and S fibers

e. Nerve impulses in S fibers elicited by non-sweet stimuli

f. Relationship between S fibers and cell types in the taste buds

### *a. Possible influence of diet on proportions of taste fiber types in two macaques*

A comparison between the taste fibers of the related, *M. mulatta*, suggests that diet differences likely are reflected in their gustatory systems. *M. mulatta *is largely a vegetarian, while *M. fascicularis*, the crab eating monkey, also feeds on littoral species cf. [24]. One prediction, based on diet differences, is that, while it is important for *M. mulatta *to be able to monitor sodium content in its diet, this may be less important for the *M. fascicularis*, because lack of sodium is not a problem for a species living along the sea. This suggestion is supported by the small proportion of N fibers in *M. fascicularis*, (15%), as compared to the 40% in *M. mulatta*. The 15% Na fibers in *M. fascicularis *parallels the 20%, based on analysis of 14 stimuli in 25 fibers, presented by Sato in the same species [25]. Otherwise there were no major differences in fiber proportions, specificity or maximum response (measured as nerve impulse frequency) between the two macaques.

### *b. Comparison with earlier data obtained with the sweet taste modifiers miraculin and GA*

In humans miraculin adds sweetness to all acids and GA blocks all kind of sweetness. We have used miraculin and GA to resolve how taste is coded in peripheral nerves, because, if these compounds exert the same taste effects in our animal models, i.e., add sweet taste to acids after miraculin or block sweetness after GA, these effects must be reflected in taste nerve responses to sweet. In the following we summarize briefly the results of several primate studies.

The first study with miraculin was done in *Cercopithecus aethiops*, an Old-World monkey [26]. The study showed that miraculin almost doubled the response to 0.03 M citric acid in recordings from the whole CT. The effects on the nerve paralleled the effects recorded from the whole human CT nerve [26]. Similar results, together with more than doubled intake of acids, were obtained in later studies of other primates: *M. fascicularis*, *C. aethiops *[27], *Saguinus m. tamarin *[28], a New-World monkey, as well as in *M. mulatta *[29]. This suggested that single fiber recordings from non-human primates could shed light on how sweetness is coded in human taste nerves.

The first single fiber study of rhesus monkey, *M. mulatta*, using miraculin showed that fibers responding to sweet, responded also to acids after miraculin [30]. Otherwise there was no difference in the nerve responses recorded before and after miraculin. After miraculin the monkeys more than doubled their intake of acids, which paralleled the results of adding sucrose to the acids. The study concluded that the increased liking of sour was caused by a response in S fibers, not by a suppression of the response to sour compounds in H fibers [30]. This finding corroborates human sensory data which show that there is no change of intensity of sourness of acids, only an increase of sweetness [31].

Corroborative data were obtained in another primate, the marmoset, *Callithrix j. jacchus*. Thus, following miraculin application to the tongue, the marmosets consumed acids more readily than before and S fibers responded to acids, although they showed no response to acids before. Once again, miraculin exerted no effects on the responses in non-sweet fibers.

As mentioned above, GA blocks sweet taste on the human tongue [32-34]. In human CT nerves GA abolished or diminished the response to sweeteners but not the response to non-sweet compounds [35,36]. Unfortunately, GA does not suppress sweet taste in monkeys as well as in all non-primates tested, although a number of earlier studies have suggested this [27]. On the other hand, in the phylogentically to human closely related chimpanzee, CT nerve recordings showed that GA abolished the response to sweet in S fibers, while it had no effect on the responses to any stimulus in non-S fibers. Behavioral data paralleled and supported the electrophysiology [37-39]. When the effect of miraculin in combination with GA was tested on a few chimpanzee S fibers, the miraculin-induced S fiber response to acids was abolished. This parallels the observation in humans that GA removes the sweetness induced by miraculin on sour compounds.

Finally, S fiber responses have and can be used to assess sweetness of new compounds, as for example, brazzein, a sweet fruit protein, in which we substituted of one or more of its amino acids. Thus, we have used monkey S fiber recordings to determine changes of sweetness of 25 mutants of brazzein. The results showed a high positive correlation(r = 0.78) with the results of assessment of sweetness of the same brazzein mutants by a human sensory panel [40,41].

In summary, the results with taste modifiers and sweeteners link the sweet taste quality to fibers of the S-cluster in all non-human primates tested. These results all support the hypothesis that activity in S fibers translates into hedonic positive responses and creates a taste that with human terminology is best described as sweet [42-46].

### *c. Parallel between lactisole results in homo and M. fascicularis*

Here we used the same lactisole concentration as the one that suppressed sweet taste in humans [17,19]. This shows that lactisole acts within the same concentration range in *M. fascicularis *as in homo.

Furthermore, the amount of suppression of each sweetener paralleled in human and in the behavior of *M. fascicularis*. The behavioral data in Figure 1 showed most suppression of saccharin and sucrose, while aspartame and acesulfame-K occupied the middle ground followed by SC 45647. In our taste panel the intensity of saccharin and sucrose was also most suppressed. Aspartame and brazzein lost about 50% of their sweetness and SC45647 was the least suppressed among the sweeteners we tested (data not shown). This order is the same as reported by Schiffman [17].

Finally, in *M. fascicularis *and humans lactisole does not significantly affect the intensity of salty, sour and bitter compounds [19]. The responses in the N, H and Q clusters of Figure 2 and Figure 4 support this conclusion. This suggests that lactisole affects the sense of taste of *M. fascicularis *and human in a similar way.

### *d. The relationship between the T1 receptors, lactisole and S fibers*

Figure 2 and 4 show that lactisole decreased the responses to sweeteners in S fibers. The literature suggests that lactisole docks to a binding pocket within the transmembrane domain (TMD) in the human T1R3 receptor [47-50]. The docking interferes with the response to sweeteners. According to Li, this TMD region, consisting of 10 residues, is the same in homo and apes, but A733 is replaced with V733 in rhesus monkey and baboon [51]. The change is probably the same in *M. fascicularis *and is apparently not preventing the effect of lactisole.

At this point it is not known if and how lactisole affects sweet taste in New-World monkeys, but the difference between human and New-World monkeys' TMDs is larger than between Old-World monkeys and humans as suggested by data from the squirrel monkey in which T735 and I739 replace A735 and T739 [51]. It is presently not known how far into the evolutionary tree the effects of lactisole reach, but it does not affect sweet taste of rats [52,53]. The elucidation of this might give further information on the nature of the T1R receptors.

The continuous trace of impulses in Figure 5 demonstrates that there was no delay of the suppression by lactisole on the S fiber response to sweet. This suggests that the inhibition a lactisole on the TMD region occurs basically at the same time as the binding of the ligand to its site on the extra cellular part of the receptor. In some experiments we used brazzein and recorded a strong suppression of S fiber responses. However, we also noticed that after the lactisole mixture was rinsed away, a response was recorded. We interpret this as the result of a stronger binding to the receptor by brazzein than by lactisole. This interpretation is supported by the psychophysical observation that the sweetness of brazzein lingers also when the tongue is rinsed with water.

To summarize, our data suggest that the suppression of sweet is the result of lactisole interfering with the TMD of the *M. fascicularis *T1R3 and that the sensory effect is conveyed in S fibers.

### *e. Nerve impulses in S fibers elicited by non-sweet stimuli*

Some S fibers respond to non-sweet compounds, although the response is less than to sweet. Figure 2 and 4 show this. If these responses originated from non-sweet receptors, there should be no suppression by lactisole, because lactisole blocks only the sweet receptor T1R3. Figure 4 confirms this conclusion. Consequently, these impulses are not caused by stimulation of sweet receptors. On the other hand, these responses occurred in S fibers. If the impulse frequency is below sensory threshold it will give no taste, if above, it should. Then they should, according to our theory, give rise to a sweet taste. This presents an apparent contradiction. In the following we present possible explanations.

It is well known that the cells of the taste buds are rapidly turned-over, e.g., [54]. One consequence of the continuous turnover is a need to reestablish connections between S fibers and T1R bearing TRCs. Thus, it is not improbable that an S fiber in search for the appropriate cells to synapse with, forms temporary connections with non-sweet TRCs, because initial hyperinnervation, followed by degeneration until normal connections are established, is a general feature in generation and regeneration. In the TB, mismatching connections degenerate, while the "right" ones remain, but before this happens, an S fiber could respond to non-sweet stimuli.

It is also possible that the compounds that elicited these responses have a sweet side taste to monkeys that is absent to humans. This is supported by the finding that taste fibers of chimpanzee are significantly more narrowly tuned to human taste qualities than those of monkey. The fact is that there are almost no S fibers responses to non-sweet stimuli in chimpanzee CT fibers and therefore most likely also in human taste nerves [38,39,55,56]. Thus these non-sweet compounds may have a hedonically positive side taste to *M. fascicularis *that it does not share with chimpanzee or human.

### *f. Relationship between S fibers and cell types in the taste buds*

Besides undifferentiated peripheral cells and basal cells, it is generally thought that the mammalian taste bud contains three major types of cells [57-59]. The largest group consists of glia cells, labeled type I cells by most investigators. They show no synaptic structures. The second type, labeled type II cells, is the TRCs. Some of these bear either T1R or T2R receptors and contain many of the constituents of the intracellular transduction components, such as gustducin and could be expected to synapse with nerves. However, they show no or few synapses with nerve endings in monkey [60] as well as in mouse and rat [57,58,61-70].

It seems that the "missing" presynaptic-like structures instead are present on a third cell type, type III. Some investigators claim that type III cells serve as the intermediate cells and receive input from more than one type II cell [71]. Thus, based on recordings from tongue slices with taste buds or patch clamp recordings of individual taste bud cells, they report that type III cells responded to many taste qualities [72,73]. If this is the only manner that taste fibers are activated, the results should be that S fibers respond to several taste qualities,

However, we and other investigators have recent data that suggest that TRCs directly can activate taste fibers, by-passing the type III link [74,75]. We found in mice with a combination of genetic, morphological, behavioral and in vivo and in vitro electrophysiological techniques that adenosine 5'-triphosphate (ATP) released from the TBs could serve as a transmitter in the TB and that knocking out the receptors for ATP, the ionotropic purinergic receptors, (P2X_2 _and P2X_3_), eliminated the taste nerve response and strongly decreased the behavioral response to sweet and bitter [74].

Later Yoshida et al. added more support to this mechanism, when they identified gustducin in the TRCs containing ATP [75]. (Gustducin is not present in type III cells). Further, they showed that the amount of ATP increased in a firing rate-dependent manner to stimulation with saccharin, quinine or glutamate. These findings suggest that the TRCs directly can activate taste nerves without involving any type III cells. This would allow a direct coupling between the T1R receptor bearing type II cells and S fibers.

Furthermore, a recent study in mice shows that breadth of tuning between CT fibers and fungiform TRCs was not significantly different [76]. This would not have been the case if information from several types of taste receptors converged on the same CT fiber, because then the breath of tuning for fibers would differ from that of taste cells. The only way to explain this is a more or less a one-to-one connection in regard to taste quality between TRCs and taste fibers.

Further support that taste qualities are conveyed separately, is offered by a recent study, which showed that neurons in the solitary tracts (NTS) respond selectively to bitter [77]. Even more interesting is that some of these NTS cells responded, not only uniquely to the bitter taste quality but also, within the bitter taste quality, to only one of the bitter stimuli used [77].

The above, does not refute an important role of the type III cells, because there are other neuropeptides and transmitters within taste buds and the mechanisms observed by [72,73] may play a modulatory role or may be crucial for intragemmal communication among the different types of cells in the TB as suggested by, for example, [78-81].

In summary, the data presented here suggest that the S and Q fiber clusters give rise to the sweet and bitter taste qualities respectively. Our data in regard to sweet is particularly strong and has withstood repeated tests in many species.

## Competing interests

The authors declare that they have no competing interests.

## Authors' contributions

TC collected behavioral data, conducted the human taste experiments and participated together with YW in the statistical analyses and production of illustrations. YW was responsible for the software development and data recording during electrophysiological experiments and participated together with TC in the statistical analyses and the production of illustrations. VD shared with GH the surgery and single fiber dissections. GH planned the project, did surgery, oversaw the data collection and processing, planned the illustrations and wrote the manuscript. AK served during the acute experiments with producing stimuli and other necessities. TR constructed the hardware for recordings, built the stimulation equipment and maintained and renewed it. GH planned the project, did surgery, oversaw the data collection and processing, planned the illustrations and wrote the manuscript.
